# Chronic graft-vs-host disease in 2016: a major challenge and an opportunity

**DOI:** 10.3325/cmj.2016.57.1

**Published:** 2016-02

**Authors:** Dražen Pulanić, Lana Desnica, Radovan Vrhovac, Damir Nemet, Daniel Wolff, Hildegard Greinix, Steven Z. Pavletic

**Affiliations:** 1 Division of Hematology, Department of Internal Medicine, University Hospital Center Zagreb, Zagreb, Croatia; 2 University of Zagreb School of Medicine, Zagreb, Croatia; 3 Faculty of Medicine Osijek, Josip Juraj Strossmayer University of Osijek, Osijek, Croatia *dpulanic@yahoo.com*; 4 Department of Internal Medicine III, University of Regensburg Medical Center, Regensburg, Germany; 5 Division of Hematology, Medical University of Graz, Graz, Austria; 6 Graft-versus-Host and Autoimmunity Section, Experimental Transplantation and Immunology Branch, National Cancer Institute, National Institutes of Health, Bethesda, MD, USA

Despite major advances in the treatment of hematological diseases over the last decades, allogeneic hematopoietic stem cell transplantation (alloHSCT) still remains the only curative option for many of them. According to the recent survey of the European Society of Blood and Marrow Transplantation (EBMT), nearly 15 000 alloHSCT are currently performed each year across Europe and EBMT-affiliated countries (1).

Approximately 50% of these patients will develop a major late complication – chronic graft-vs-host disease (cGVHD), a multi-organ allo- and auto-immune disorder affecting the skin, lungs, mouth, liver, eyes, joints, and gastrointestinal and genital tracts (2-4). As a multisystem disease in these long-term survivors after alloHSCT, it presents with a number of heterogeneous clinical manifestations requiring a multidisciplinary approach both in its diagnosis and treatment.

Chronic GVHD can last for many years causing severe medical, social, and quality of life problems, as well as significantly impacting health-related costs and health care management. A recently published report from the Center for International Blood and Marrow Transplant Research has identified a clear increase in the incidence of cGVHD from 1995 to 2007 due to the more frequent use of peripheral blood graft instead of bone marrow, unrelated donors, and older recipients (5). In addition, because of increasing safety of alloHSCT and better supportive care, there are more long-time survivors after alloHSCT and hence more patients are at risk of developing cGVHD (5).

cGVHD has been much better characterized using internationally recognized diagnostic criteria and scoring measurements since the first National Institutes of Health (NIH) Consensus classification was developed in 2005 and prospectively validated (3,6-10). However, there are still plenty of issues in addressing challenges posed by cGVHD to clinicians and academic researchers. In June 2014, the second cGVHD consensus conference was held at the National Cancer Institute (NCI), NIH, USA, attended by 250 participants from all over the world. A number of updated evidence-based recommendations were proposed at this conference and are detailed in the six new consensus papers published during 2015 in the Biology of Blood and Marrow Transplantation journal (4,11-15). These new recommendations should further advance cGVHD clinical research related to diagnosis, staging, histopathology evaluation, biomarkers, response criteria, design of clinical trials, estimating prognosis, guiding therapeutic decisions, ancillary and supportive care, as well as standardizing documentation.

The German-Austrian-Swiss cGVHD consortium assessed the usefulness of the NIH criteria in routine clinical practice and reported high rates of acceptance for definitions of cGVHD, as well as overall and organ-specific NIH cGVHD severity scoring among the vast majority of participants (16). An international survey of the EBMT-NCI cGVHD Task Force conducted in 2013 confirmed substantial national and international support for use of the NIH Consensus criteria in everyday clinical practice (17). With the goal to implement the newest diagnostic criteria and clinical standards for cGVHD in Croatia, a multidisciplinary clinical infrastructure for cGVHD was established at the University Hospital Center Zagreb in the middle of 2013, supported by the Unity Through Knowledge Fund project entitled “Clinical and Biological Factors Determining Severity and Activity of Chronic Graft-Versus-Host Disease after Allogeneic Hematopoietic Stem Cell Transplantation” funded by the World Bank and Croatian Ministry of Science, Education and Sports. Until now, 53 cGVHD patients have been prospectively evaluated in Zagreb by a multidisciplinary team of subspecialists in hematology, dermatology, dentistry, ophthalmology, physical therapy, pulmology, gynecology, nutrition, neurology, and other specialties when needed, with a collection of standardized demographic, clinical, laboratory, histopathology, and imaging data. Moreover, additional 43 transplanted patients without cGVHD were evaluated by Zagreb's team to serve as controls. This approach improved the consistency of assessment and treatment of patients with cGVHD and also validated the care-models and standards put forward by the internationally developed NIH criteria. This initiative is also playing a pivotal role in promoting interdisciplinary and international collaboration, stimulating education, exchange, and networking of researchers and clinicians, as well as the development of several scientific subprojects on this often devastating chronic disease.

Therefore, there has been significant research activity and renewed interest in cGVHD in the world over the last decade. It is likely that the current better characterization of cGVHD, standardization of research tools, and numerous new treatment opportunities will lead to improved clinical outcomes, such as clinical symptoms, function status, quality of life, morbidity, and survival.

However, not a single agent has yet been approved for cGVHD prevention or treatment, neither by the US Food and Drug Administration (FDA) nor by the European Medicines Agency (EMA). The current standard front-line steroid therapy has a 50% failure rate with significant toxicity, and there are no standards for second-line (and beyond) therapeutic options.

The focus now in this field is on further in-depth study of the biology of cGVHD, developing and validating new biomarkers, and pursuing clinical trials of new agents that would eventually develop pathways to their FDA and EMA regulatory approvals. The aim is to develop more effective, less toxic, and more targeted treatments that will not interfere with the beneficial graft-vs-tumor effects. To advance this field and break the 30-year-old suboptimal treatment paradigms, the goal is that each cGVHD patient should be either treated in a clinical trial or at least should be documented within a registry capturing essential clinical data on the course of cGVHD. The tools and opportunities to harness cGVHD and create better and safer alloHSCT are in our hands right now. This issue of the *Croatian Medical Journal* presents several excellent articles that are a product of these enhanced international collaborations and efforts by teams across Europe to address cGVHD.

**Acknowledgment** This work is supported by the Unity Through Knowledge Fund project entitled “Clinical and Biological Factors Determining Severity and Activity of Chronic Graft-Versus-Host Disease after Allogeneic Hematopoietic Stem Cell Transplantation.” The opinions expressed here are those of the authors and do not represent the official position of the National Institutes of Health or the US Government.
